# Simulations of x-ray absorption spectra for CO desorbing from Ru(0001) with transition-potential and time-dependent density functional theory approaches

**DOI:** 10.1063/4.0000135

**Published:** 2022-01-13

**Authors:** Gabriel L. S. Rodrigues, Elias Diesen, Johannes Voss, Patrick Norman, Lars G. M. Pettersson

**Affiliations:** 1 Department of Physics, AlbaNova University Center, Stockholm University, SE-10691 Stockholm, Sweden; 2 SUNCAT Center for Interface Science and Catalysis, SLAC National Accelerator Laboratory, 2575 Sand Hill Road, Menlo Park, California 94025, USA; 3 Department of Theoretical Chemistry and Biology, School of Engineering Sciences in Chemistry, Biotechnology and Health, KTH Royal Institute of Technology, SE-106 91 Stockholm, Sweden

## Abstract

The desorption of a carbon monoxide molecule from a Ru(0001) surface was studied by means of X-ray Absorption Spectra (XAS) computed with Transition Potential (TP-DFT) and Time Dependent (TD-DFT) DFT methods. By unraveling the evolution of the CO electronic structure upon desorption, we observed that at 2.3 Å from the surface, the CO molecule has already predominantly gas-phase character. While C 1s XAS is quite insensitive to changes in the C–O bond length, the O 1s excitation is very sensitive with the π* coming down in energy upon CO bond stretching, which competes with the increase in orbital energy due to the repulsive interaction with the metallic surface. We show in a systematic way that the TP-DFT method can describe the XAS rather well at the endpoints (chemisorbed and gas phase) but is affected by artificial charge transfer and/or incorrect spin treatment in the transition region in cases like CO, where there are low-lying π* orbitals and large exchange interactions between the core 1s and valence-acceptor π* orbitals. As an alternative, we demonstrate by comparing with experimental data that a linear response approach using TD-DFT employing common exchange-correlation functionals and finite-size clusters can yield a good description of the spectral evolution of the 1s → π* transition with correct spin and gas-to-chemisorbed chemical shifts in good agreement with experiment.

## INTRODUCTION

A fundamental problem in surface science, underlying all surface reactions relevant for catalysis, is the exact nature of the bond between a given adsorbate and the surface. Bond formation relies on hybridization of discrete molecular or atomic orbitals with the electronic bands of the substrate, in the case of chemisorption, as well as long-range dispersive forces in physisorption. Due to its site-specific excitation mechanism and sensitivity to the local valence electronic structure at the core-excited atom, core-level spectroscopies have allowed highly detailed investigations of adsorbate–surface bonds.^1,2^ The combination of modern intense femtosecond optical laser systems with free-electron x-ray lasers has made it possible to follow the progress of chemical reactions in real time with picosecond resolution. This has allowed experimental probing of short-lived species in surface reactions, giving invaluable information about entire chemical processes, such as desorption, transition states, and bond-breaking and formation.^3–7^ These ultrafast time-resolved x-ray spectroscopy experiments show enormous potential for identifying reacting atomic and molecular species and their local electronic structure with large sensitivity and selectivity.^8–10^ Specifically, x-ray absorption spectroscopy (XAS) provides information about unoccupied states of molecular systems and reveals details about the local chemical environment of given species.^11–13^ Therefore, XAS can not only identify specific adsorbates on surfaces, but also differentiate between the same species adsorbed at different sites, as well as resonance formation due to a change in the local electronic environment.

However, often the experimental results can be hard to interpret, e.g., if there are numerous species at the surface or if transient states are probed^3,4,7^ for which no steady-state reference is available. Thus, theory and accurate computational methods are needed to fill the gaps and give necessary information for an extensive description. Furthermore, computational studies can predict experimental behavior, and, when performed ahead of the experiment, provide an expectation of what to look for and where.

Desorbing adsorbates pose a specific challenge for the calculation of core-level spectra, since often different approximations are needed to treat the substrate compared to the gas-phase molecule. Especially for a metallic substrate, a periodic slab calculation or an extended cluster model is needed to accurately reproduce the metallic band structure and fully describe the chemisorbed system, which is usually only feasible with GGA-level DFT. The coupling between the discrete atomic or molecular levels and the band structure of the substrate broadens the spectroscopic features and limits the possible experimental resolution, which allows for applying more approximate computational techniques. The higher resolution that is achievable for gas-phase molecules, on the other hand, might require highly accurate treatment of electronic exchange and correlation, which necessitates hybrid functionals or high-level quantum chemical methods that are far too computationally expensive to use in a periodic slab calculation. For each of these end-points, chemisorbed and gas phase, accurate, and reliable computational spectroscopic techniques have been developed and are readily applicable. However, with the introduction of ultrafast pump-probe techniques following, e.g., desorption from a surface into gas phase, it becomes necessary to investigate how these computational approaches perform also in the intermediate range between chemisorbed and gas phases.

Among the most widespread computational techniques to calculate core-excited absorption spectra is the transition potential (TP) method within the Kohn–Sham (KS) orbital formalism.^14–16^ This is an approximation to the Slater transition-state approach^17,18^ to excitation energies, in which half an electron is moved to the excited orbital. Approximating the excitation energy as the difference in orbital energies between these two half-occupied states can be shown to be correct to second order in the response to the change in occupation.^17–19^ However, the original approach of Slater requires state-by-state calculations, variationally optimizing states with a half-occupied initial orbital and successively placing the excited half electron in higher and higher excited orbitals. Computing XAS typically requires hundreds of states to properly describe valence, Rydberg, and continuum excitations, which makes a state-by-state calculation approach unwieldy. The half-core-hole TP approximation builds on the fact that the higher excited states interact only weakly with the molecular ion core, and neglecting the presence of the excited half electron is thus a good approximation.^19^ The full XAS spectrum is then obtained from a single variational determination of the density in the presence of the half-occupied core-level. More strongly interacting valence-excited states can be corrected in terms of energy by explicit ΔKS calculations of a few of the lowest core-excited states.^20^ The TP-DFT approximation has been successfully applied to surface adsorbates^14,21–30^ as well as gas-phase molecules,^14,29–34^ but the transition from chemisorbed to gas phase is less well investigated.

CO desorption from Ru(0001) has been measured at the O *K*-edge and modeled with TP-DFT using a cluster model of the surface with focus on the spectroscopic development as the molecule entered the precursor state prior to desorption/readsorption.^3,6^ Excitation at the O *K*-edge of CO leads to a strongly vibrationally broadened π* profile, while the C 1*s* → π* is very sharp which allows additional features to be resolved.^7^ TP-DFT XAS calculations using a cluster model and Gaussian basis sets were found not to converge to the proper gas phase limit, except at unphysically large distance (see results below), while the same approach using periodic boundary conditions and a numerical grid^16^ resulted in a better description.^7^ The origin of the improper description of the spectrum upon desorption using TP-DFT in combination with the cluster model is the presence of the half-core-hole, which gives the molecule a formal +0.5 charge. In the gas phase, this is simply a computational trick as no charge can be transferred. When strongly chemisorbed, there is already charge transfer from the metal through the chemical bond and the core-hole does not significantly affect that. Thus, TP-DFT describes the end-points very well. However, the presence of the core-hole as the molecule desorbs leads to charge transfer from the metal also at distances where the molecule is no longer interacting with the surface, but where weak orbital overlaps still provide a channel to screen the positive charge. Thus, to overcome this situation, it becomes necessary to explore approaches that are based on the response of the *ground-state* wave function to the external electromagnetic field, without the need to introduce a half-occupied core-hole.

Finite-size cluster models combined with local basis-sets, where the cluster model is constructed in such a way that most chemical environment effects are captured around the atom, molecule, or bond that one wants to study, have a long history.^35–40^ This is usually a reasonable approximation as chemical bonds are rather local.^41^ Metallic clusters can become an attractive approach, because with local basis sets it is in principle computationally feasible, still with limitations to basis set size, to use multi-reference wave function (WF) excited-state methods or high-level DFT functionals, such as global (GH) and range-separated (RSF) hybrid functionals, which in principle give a better description of the local chemical bond and electronic transitions due to the inclusion of some amount of exact Hartree–Fock (HF) exchange.

One of the most computationally efficient and widespread approaches to excited-states is linear-response TD-DFT, where the response of the electron density of a ground-state Kohn–Sham system to a time-dependent electromagnetic field is determined. However, in terms of absolute core excitation energies, TD-DFT is very dependent on the choice of exchange-correlation (xc) functional,^42–46^ making the onset of spectra dependent on the functional applied. Fortunately, it is usually true that the errors are systematic,^47^ making it possible to reproduce quantitatively, e.g., chemical shifts across similar systems. Therefore, one can directly compare experimental and computed spectra by shifting the calculated one to fit the experimental data for one specific reference system.

Mostly, TD-DFT is used for valence-electronic excitations as these are the first available (lowest energy) excitations of a system, while the direct computation of core-valence excitations would require an enormous number of states in the calculation. However, due to the weak coupling between core and valence states and the strong localization of the former, it is generally a good approximation to separate the core region of the Hamiltonian matrix and to perform only the diagonalization of this part of interest. This is the core-valence separation (CVS) approximation.^48^ The CVS linear-response TD-DFT approach has already been used with success for organic molecules and molecular systems in general, but, to the best of our knowledge, it has never been employed to calculate x-ray spectra of surface species using metal clusters as models.

In the present work, we apply linear-response TD-DFT, employing different exchange-correlation functionals to investigate the use of metal clusters of different sizes to simulate time-resolved x-ray free-electron laser (XFEL) experiments monitoring CO desorption from a Ru(0001) surface using XAS.^3,7^ Comparison is made with a more traditional TP-DFT periodic XAS calculation as we have observed that TP-DFT can have an improper behavior at intermediate desorption distances. We assign this problem to artificial charge transfer which we will discuss in more detail below. Therefore, our main objective is to evaluate if a linear-response approach like TD-DFT can be used as alternative to TP-DFT for simulating surface chemistry spectra from pump and probe experiments. We emphasize that this process is a good case of study as it was already studied experimentally for C1s^7^ and O1s^3^ XAS, providing reliable data that we will use as our main reference.

## METHODS

### Ground state

**FIG. 1.**
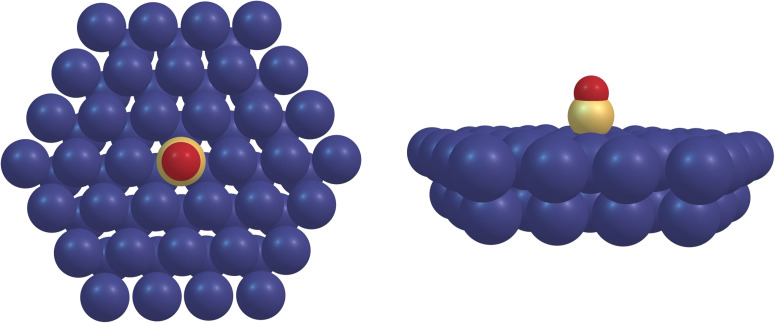
Ru_64_ cluster structure from the chemisorbed ground-state extracted from the PBC calculations with B3LYP optimized rCO and rRu-C parameters.

Finite-size cluster models were obtained from an optimized structure of a CO molecule chemisorbed on a Ru(0001) top site calculated using Periodic Boundary Conditions (PBCs) and the RPBE functional. Five cluster sizes were cut from the solid-state structure, containing 10 (Ru_10_), 19 (Ru_19_), 31 (Ru_31_), and 64 (Ru_64_) Ru atoms. For the present purposes, it was deemed sufficient to include the first two layers as we are dealing with unoccupied states that mainly are localized on the CO molecule (see Fig. 4 for the spatial extent of the dominating transitions). The clusters were constructed preserving the symmetry along the Ru–CO bond axis. On each cluster, the equilibrium geometry was obtained by optimizing the Ru–CO and the C–O bond lengths while freezing all other degrees of freedom. This was done using one GGA functional, RPBE,^49^ and four hybrid functionals, PBE0,^50^ B3LYP,^51^ CAM-B3LYP,^52^ and CAM-B3LYP(100%).^53^ We note that although CAM-B3LYP is not usually used for geometry optimizations, it was used here for sake of consistency. The Ru_64_ cluster is shown in Fig. 1 as an example.

From the equilibrium structures, coordinate-driven potential energy surfaces (PESs) following the CO desorption were built fixing the Ru–CO bond length (rRu-C) from equilibrium to 10.0 Å in eight steps where rRu-C = 1.90, 2.00, 2.10, 2.30, 2.50, 2.70, 4.00, and 10.0 Å. In all these new cluster structures, the C–O bond length was the single optimized parameter. The calculated PESs can be seen in Figs. S1–S3 in the supplementary material. The converged wave function from each one of these PES points was used as the starting point for the subsequent TD-DFT calculations.

All cluster ground-state calculations along the PESs were carried out using the ORCA software^54^ applying Grimme's atom-pairwise dispersion^55^ with Becke–Johnson damping (D3BJ)^56^ and the triple-zeta def2-TZVPP basis set^57^ for Ru_10_ and Ru_19_ while def2-TZVP^57^ was used for Ru_31_. In the case of the Ru_64_ cluster, due to the large size of the systems, no PES was fully built and the rC-O optimized parameters from Ru_31_ PESs were used in the TD-DFT calculations. For Ru atoms, these basis sets are employed with a 28 core-electron effective core potential (ECP). Therefore, only the 16 outermost electrons of each ruthenium atom are considered as valence and included in the calculations. Computational speed-ups were achieved using the resolution of identity (RI),^58^ in conjunction with the def2/J auxiliary basis set provided in ORCA, and the chain of spheres (COS)^59^ approach for the Coulomb and exchange parts of the Fock matrices, respectively. More details of the calculations, including level shifts and integration grids for better convergence, can be found in the supplementary material.

Periodic boundary condition (PBC) calculations were performed using a 2 × 2 4-layer slab setup in Quantum ESPRESSO.^60,61^ The RPBE functional was used, and 4x4x1 *k*-points were used for structural relaxation, with the experimental lattice constant 2.7 Å. Ultrasoft pseudopotentials were used for treating the atomic core electrons.^62^ Starting from the optimized slab, the desorption structures were obtained by increasing the Ru–C distance and then optimizing the C–O bond length, just as for the cluster calculations. Further details can be found in the supplementary material.

### Excited states

The XAS spectra were computed using two different excited-state methods implemented in three different quantum chemistry packages. The aim was to compare a solid-state code using the half-core-hole/TP approximation to a linear response theory framework performed in the cluster calculations within the TD-DFT formalism, as well as TP-DFT within a cluster framework.

TD-DFT calculations were carried out using ORCA employing five different exchange-correlation functionals: one GGA functional (RPBE), two “conventional” global hybrid (PBE0, B3LYP), and two range-separated hybrid [CAM-B3LYP, CAM-B3LYP(100%)] functionals. The main feature of RSFs is that, instead of using a fixed fraction of DFT and HF exchange, they have different contributions according to the spatial distance between two integration points.^66^ This makes this type of functional a good choice for excited-state calculations where the electronic density can be displaced across large interelectronic distances, especially regarding XAS. The CAM-B3LYP (100%) is a reparameterization of the CAM-B3LYP functional where 100% exchange is used for long-range interactions.^53^ This functional has been shown to work well for XAS calculations of organic molecules and details can be found in the literature.^63–65^

The TD-DFT calculations followed the same protocol as the geometry optimizations, but with modified basis functions. For C and O atoms we employed Truhlar's minimally augmented version of the def2-TZVP (ma-def2-TZVP^67^) triple zeta, double-polarized basis sets. For the single Ru atom bonded to the CO carbon def2-TZVP(-f) basis set (with ECP) was used but without the *f* functions. Finally, all the other Ru atoms were described by the def2-SV(P) double-zeta basis set, where we removed small *s* and *p* exponents. This choice of different basis sets was necessary to obtain SCF convergence while still preserving a good description of the local Ru-CO bond. More details can be found in the supplementary material.

XAS was also calculated from the periodic slab structures using the TP-DFT approach.^14^ With this method, the spectrum is calculated from the unoccupied density of states in a DFT calculation, with a modified pseudopotential on the C atom representing one-half core hole. The unoccupied density and transition dipole moments are calculated efficiently using a Lanczos recursion Green's function technique, as implemented in the xspectra code.^68–70^ To obtain an accurate absorption onset energy, the total energy of the lowest core-excited state is calculated with a pseudopotential corresponding to a full core hole, and the difference to the ground state energy is then used to shift the spectrum obtained from the half-core hole calculation (ΔKS method). The positive charge of the excited core is compensated for by adding additional electronic charge to the system, so that the unit cell is kept neutral. To avoid interaction of the core-hole with its periodic images, the slab was increased to 4 × 4 surface atoms. This method has been shown to reproduce XAS shifts between, e.g., different adsorption sites well;^16,71^ however, it cannot give an absolute energy scale when using pseudopotentials for the core electrons, so an overall shift must be applied to all spectra for a comparison with absolute experimental energies.

When treating gas-phase molecules the single-determinant nature of a DFT reference state can lead to an incorrect absorption onset^72^ in the TP approach since core-excited molecules can exhibit significant singlet–triplet splitting due to the exchange interaction between the remaining core electron and the excited one. This occurs if the excited state has significant spatial overlap with the core-orbital, as is the case for CO, most notably at the C K-edge. To obtain the correct absorption onset corresponding to the singlet state, the correction scheme from Refs. 73 and 74 was applied, by implementing spin-polarized core 1*s* states to calculate the required total energy difference between anti-parallel and parallel excited electron spin. For gas phase CO, the required energies can be readily calculated by constraining the occupation of molecular orbitals; however during desorption, the spin state of the molecule cannot be controlled without introducing constraints that would give inconsistent total energies.

### TP-DFT cluster calculations

The TP-DFT calculations were performed using the StoBe-deMon DFT code^75^ with exchange and correlation from Refs. 76 and 77 and a 17-atom, three-layered cluster modeling on-top adsorption on the Ru(0001) surface. For C 1*s* excitation, carbon was described using the IGLO-III basis^78^ to better describe core-relaxation, while oxygen was described by a (6311/311/1) basis. Ruthenium was described by the 14-electron model potential of Andzelm *et al.*^79^ combined with a (2211/3111/311) Gaussian basis set. The cluster structure and CO geometries were taken from Ref. 3. For the spectrum calculations, after converging the density for the system with the half core-hole, the above basis set was extended with a diffuse (19s19p19d) basis to better cover Rydberg and continuum states. Note that the density was not reoptimized with this basis, but instead a single diagonalization was performed of the KS matrix built with the previously converged density in order to obtain an extended set of virtual orbitals in the potential from the half core-ionized system. The excitation energy was corrected by explicitly computing both the singlet and triplet 1s → π* excited states. The triplet energy (E_T_) is well described by a single determinant while the computed singlet (E_S_) is an equal mixture of the correct triplet and singlet which allows obtaining the correct singlet energy as 2 E_S_ - E_T_. This is possible since the core electrons are explicitly included in the calculation.

## RESULTS

### Experimental pump and probe spectra

**FIG. 2.**
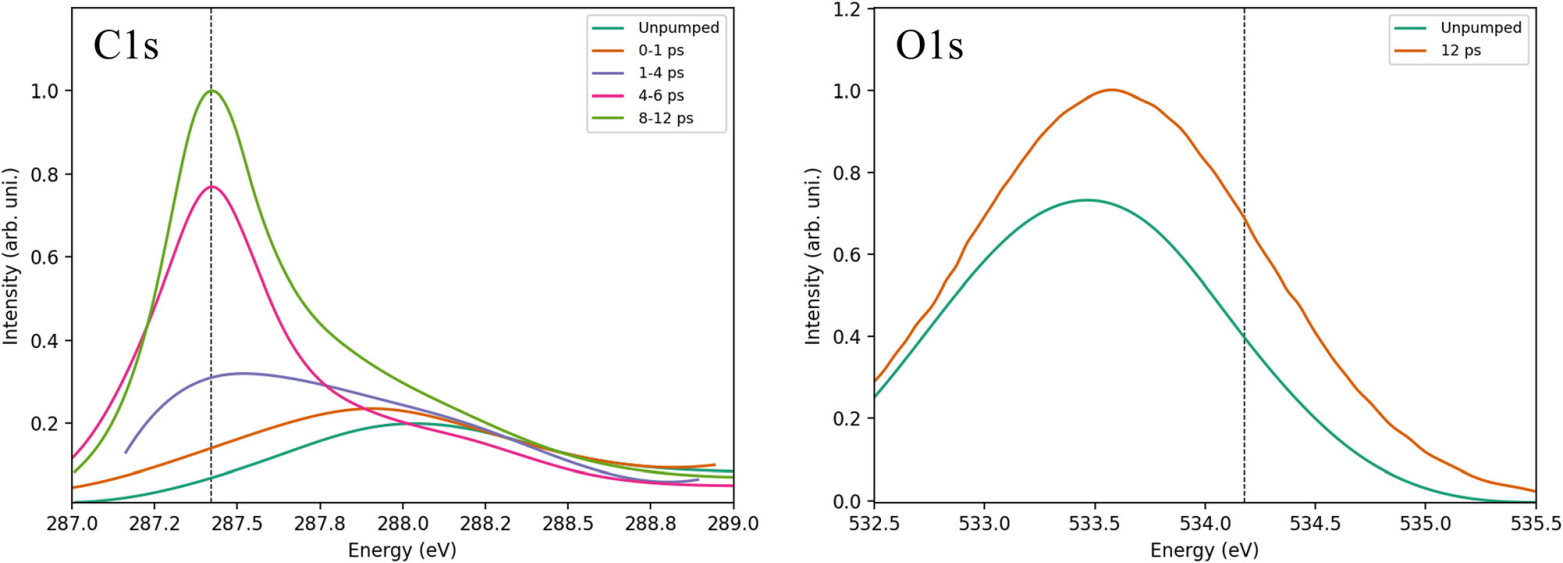
Carbon (C1s) and oxygen (O1s) *K*-edge XAS evolution in CO/Ru(0001) pump and probe experiments. Experimental data taken from Refs. 3 and 7 for C1s and O1s, respectively. The vertical dashed lines show CO gas-phase values included in the references.

For sake of comparison, the experimental carbon and oxygen K-edge spectra are reproduced in Fig. 2, where data were extracted from experimental pump and probes studies.^3,7^ The XAS in Fig. 2 shows that from the chemisorbed (umpumped) toward the desorbed CO molecule the π* peak increases in intensity and exhibits a negative shift toward the gas-phase excitation energy for C1s while the shift is positive for O1s. The reason for this different behavior between carbon and oxygen will be discussed below. We can see that in the carbon K-edge spectra around 8–12 ps after the pump, the π* peak position is essentially at a gas phase value of 287.4 eV, and the peak is sharp and well defined, characterizing a CO molecule that is desorbed from the surface. Differently, in the oxygen K-edge spectrum the π* peak has not yet converged to the gas phase energy at 12 ps. However, we can see that there is a positive shift from the unpumped to the 12 ps spectrum going toward the gas-phase value. More details of the discussion around these spectra can be seen in the original works for the oxygen^3^ and carbon^7^ K-edges.

### CO electronic structure along desorption pathway

Before the discussion of the calculated x-ray absorption spectra of the CO/Ru(0001) system, we briefly introduce the electronic structure of CO desorbing from the Ru(0001) surface, which affects the behavior of the main feature in the x-ray absorption spectra: the first peak in both C 1*s* and O 1*s* transitions. This is the most intense peak in C and O K-edge XAS of CO and corresponds to the transition from the carbon or oxygen 1*s* to the degenerate lowest-energy unoccupied orbitals (LUMO), which are the two π* orbitals formed by the antibonding combination of *p_x_* or *p_y_* orbitals from both atoms (the z-axis is taken along the molecular axis). Hence, one important structure parameter to follow along the PESs, as it is directly related to the two π* orbitals, is the C–O bond length. Figure S4 in the supplementary material contains a plot of C–O bond lengths optimized at each point of the potential energy surfaces and shows the expected behavior that the CO bond shortens upon desorption due to the increase in internal CO bond strength.

**FIG. 3.**
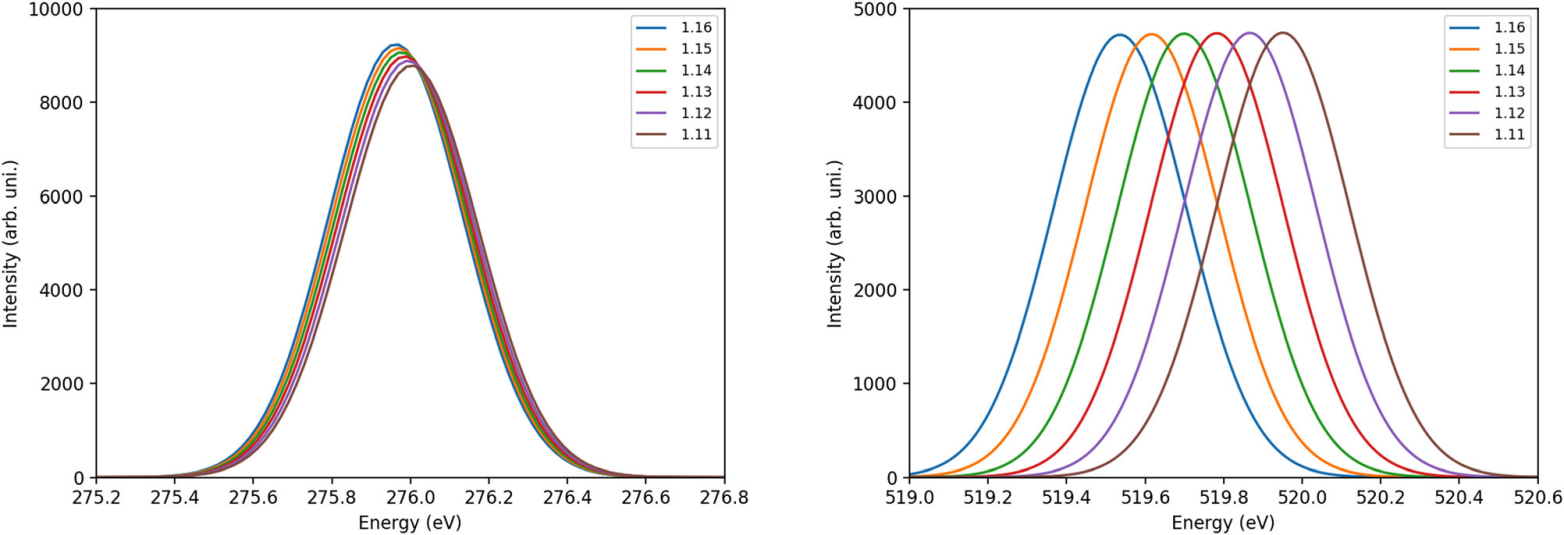
CO(g) C 1*s* (left) and O 1*s* (right) x-ray absorption spectra for different C–O bond lengths calculated with the CAM-B3LYP functional. Bond length values in the legends are in Å.

**FIG. 4.**
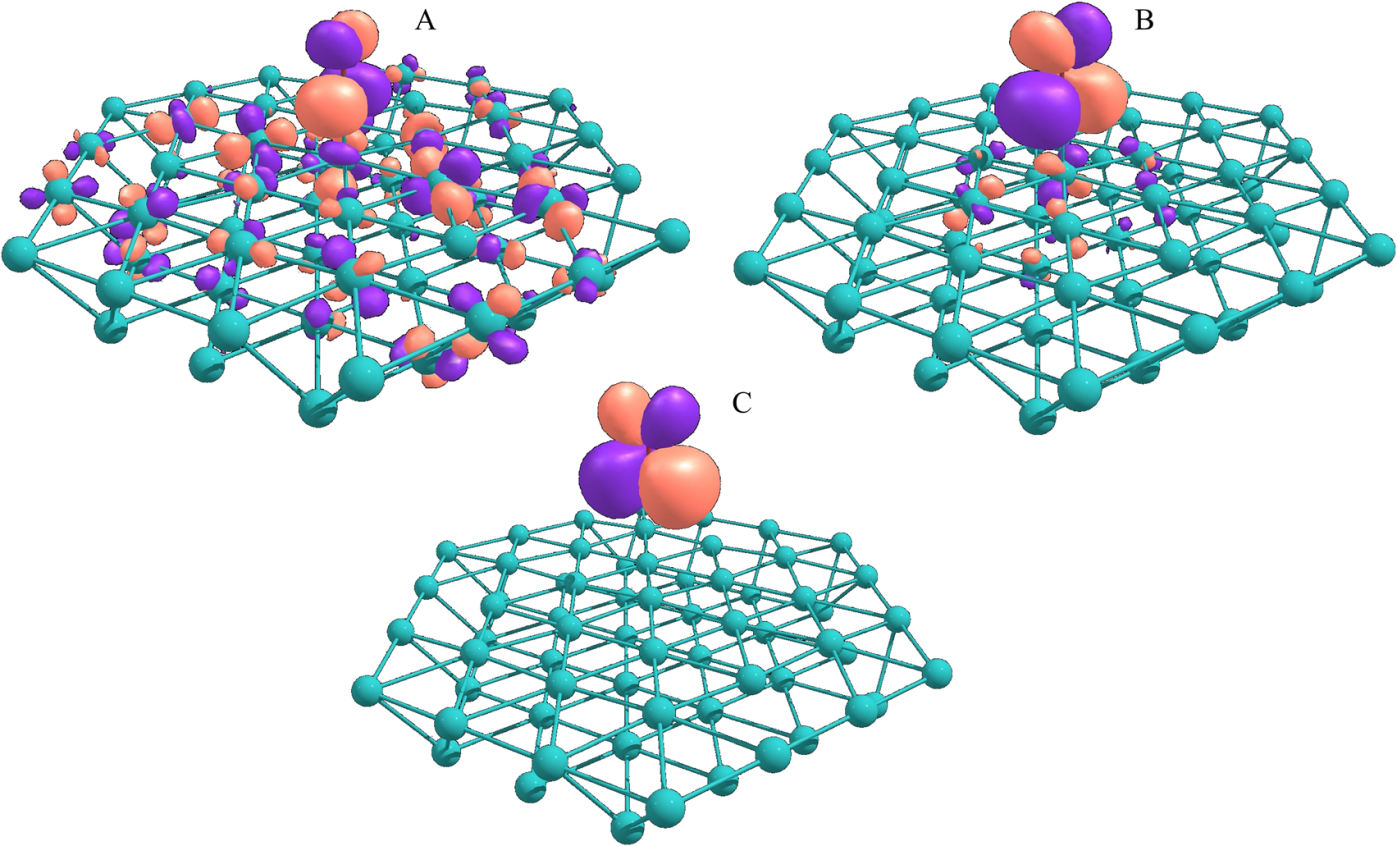
Acceptor NTOs from TD-DFT/CAM-B3LYP C 1s XAS calculations in Ru_64_ clusters for Ru–CO distances of (a) equilibrium (1.82 Å), (b) 2.30, and (c) 4.00 Å. Shown distances are with respect to rRu-C. We can see the NTOs can be easily identified as mostly CO π* orbitals.

We can remove the effect of bonding to the surface and look how the C–O bond distance impacts the π* and the XAS spectra. Figure 3 shows the C 1*s* and O 1*s* XAS spectra for gas-phase CO at different C–O bond lengths. Since the π* is an antibonding orbital, the decrease in the CO bond distance leads to an increase in the π* energy, and this is what we see in both spectra going from 1.16 to 1.11 Å. Moreover, it is immediately seen that, while the O 1*s* spectra are quite sensitive to the internal distance, the C 1*s* spectra show minimal changes. Including now the surface, Fig. 4 shows one of the two near-degenerate π* orbitals as acceptor natural transition orbitals (NTOs) from TD-DFT/CAM-B3LYP calculations for the Ru_64_ cluster. NTOs are built from a weighted sum of the canonical orbitals involved in the transition and the ones shown in the picture represent more than 99% of the contribution to the C 1*s* XAS first peak. We can see that at equilibrium the Ru(*d*)-CO(π*) interaction is repulsive due to antibonding character and, therefore, as the CO molecule desorbs from the surface the π* energy must go down, contrary to the effect of bond shortening, which increases the π* energy. Thus, we have a competition between these two effects regarding the chemical shift. Since the π* energy in the carbon spectrum is insensitive to changes in the bond length, the repulsion is the dominating effect and, as we saw in the experimental spectra, we have a negative shift from chemisorbed toward the gas phase. In the oxygen spectrum, the opposite is true, and the reduction of the repulsion is not enough to compensate for the increase in π* energy due to bond shrinkage, resulting in a positive chemical shift from chemisorbed toward the gas phase. The opposite behavior of the π* orbital upon C 1*s* and O 1*s* excitation may seem counterintuitive but is easily understood in terms of the Z + 1 approximation where the removal of one screening 1*s* electron is equivalent to increasing the nuclear charge by one unit.^80^ Thus, excitation from the carbon 1*s* into π* effectively results in NO with N–O bond distance of 1.151 Å while the oxygen *K*-edge gives CF with a bond distance of 1.272 Å, compared to gas phase CO at 1.128 Å. For C 1*s* the vertical excitation remains close to the minimum of the core-excited state (∼NO) potential energy surface (PES) with variations in the ground state C–O distance, while for O 1*s* vertical excitation goes to the steeply repulsive part of the CF PES and thus becomes very sensitive to geometrical changes.

Looking again at Fig. 4, at equilibrium the NTO is composed of CO-π* with contributions from *d* orbitals belonging to Ru atoms all over the cluster, which in solid-state corresponds to interactions with the *d*-band, forming an extensively delocalized orbital. Regarding the orbitals in Fig. 4, this remains true up to 2.30 Å, where most of the *d* character is lost and the orbital is almost pure CO-π*. Complementary to this, the ΔrC-O increments are significant only up to 2.30 Å as shown in Fig. S4, which indicates that beyond this point the molecule already has a significant gas-phase character. Figure S5 shows the same orbitals calculated with the other exchange-correlation functionals and cluster sizes. For some other cases, there is still a significant amount of *d*-orbital contribution at rRu-CO = 2.30 Å. However, at 4.00 Å, there are no significant contributions from the *d*-orbitals and the acceptor orbital is a completely localized gas-phase-like π*.

#### TP-DFT

**FIG. 5.**
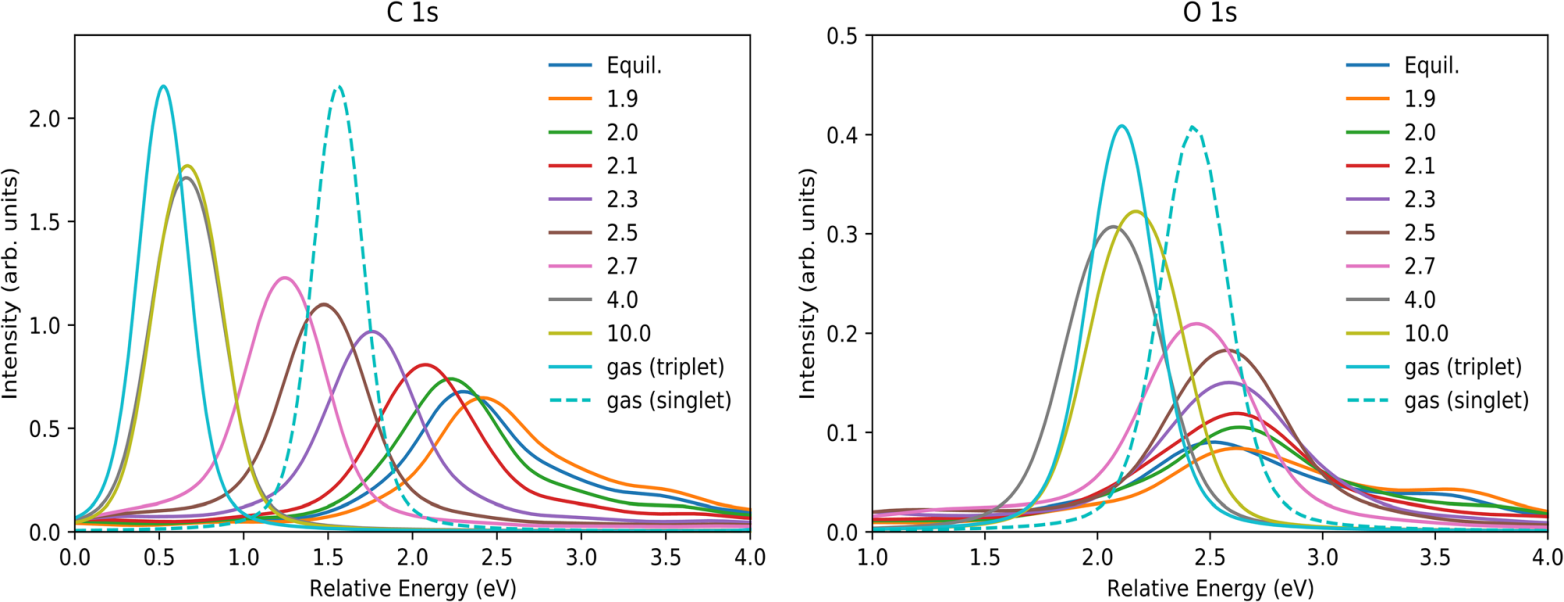
C 1*s* and O 1*s* XAS spectra calculated with the periodic model within the TP-DFT method. Energies given relative to the Fermi level.

The XAS for a desorbing CO molecule calculated for the periodic model using the TP-DFT method is shown in Fig. 5 for the C and O edges. The C peak shifts to significantly lower energies as the surface-adsorbate distance increases, while a much smaller shift is observed for O. The main change in width occurs already at 2.1–2.3 Å distance, which is expected from previous observations of rC-O and CO-π* orbitals that show these to be close to gas-phase beyond this distance. It is clearly seen that, as the molecule desorbs, the peak approaches the energy position of that calculated for a gas phase molecule with a triplet configuration after excitation. For a neutral gas phase CO molecule with a core-hole, this is indeed the lowest energy configuration; hence, it is to be expected that this is the variational minimum when charge transfer from the surface can occur. However, the open-shell singlet is the actual final state after core excitation. The spurious charge transfer from the substrate not just screens the core hole charge but leads to an incorrect spin state for the excited state. This appears to be the main cause of the large negative shift for large distances, when the slab is still present.

The computed peak offset between the (singlet) gas phase and chemisorbed is around 0.74 eV for the C excitation and 0.1 eV for O. This should be compared with experimental values of 0.59 eV^7^ and −0.75,^6^ respectively. There seems to be a systematic underestimation of the energy of the core-excited gas phase molecule compared to the adsorbed one, while the change in the peak width compares well with experiment.^7^ Compared to, e.g., shifts between adsorbate sites on a substrate, the chemical environment of the core-excited atom changes much more drastically upon desorption, so that a weaker agreement is not surprising. Absolute adsorption energies of CO on transition metals are not generally well described by GGA-level DFT,^81^ with deviations of several tenths of an eV, often also giving an incorrect site preference. This means that the electronic structure is not represented well enough to reproduce the bond formation in a quantitatively exact way. Although for the particular case of CO/Ru the RPBE adsorption energy agrees quite well with experiment,^82^ the same is not necessarily true for the core-excited molecule, and this will give an error in our adsorption onsets affecting the position of the LUMO resonance.

**FIG. 6.**
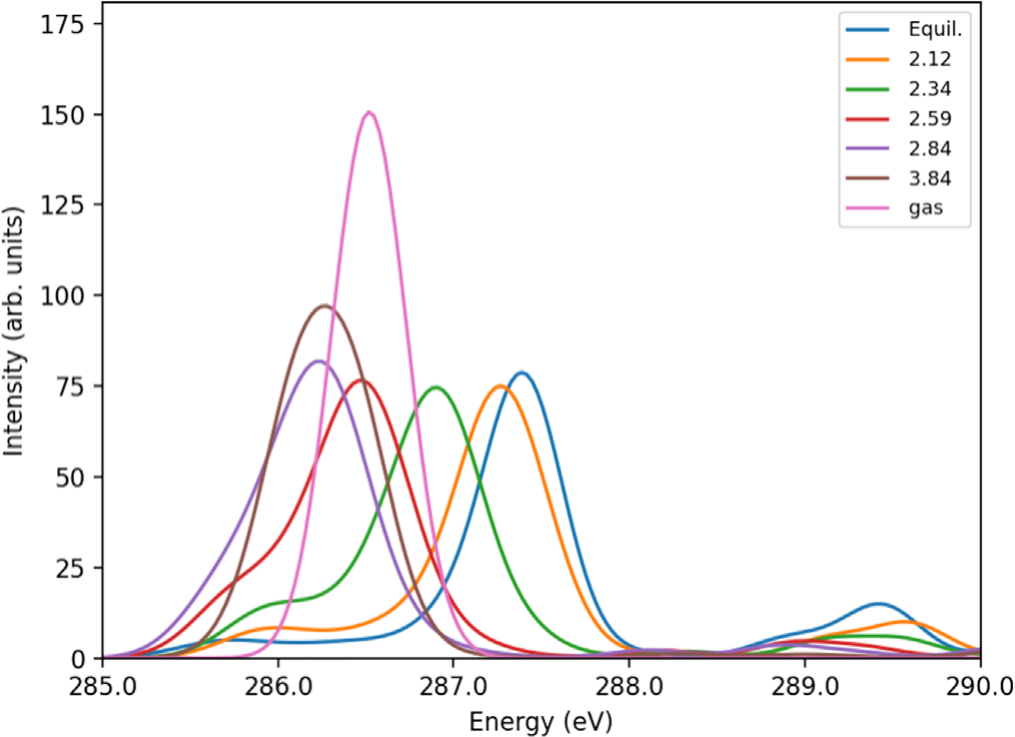
Computed TP-DFT XAS for CO at different Ru-C distances (Ångström) using the Ru_17_ cluster model showing the progression from chemisorbed (Ru–C = 1.94 Å) to desorbed at 3.84 Å. The gas phase π* at 286.5 eV is included as reference.

In Fig. 6, we show the C 1*s* spectra computed using the TP-DFT approach with Gaussian basis sets and a Ru_17_ cluster model of the surface. The shift of the C 1*s* → π* peak of chemisorbed CO compared to gas phase is in the correct direction albeit somewhat overestimated (+0.9 vs +0.59 eV) compared to experiment^7^ showing a satisfactory description of the two endpoints (chemisorbed vs gas phase). Here, it should be noted that the correct singlet excitation was used by calculating the fully relaxed core-excited state at each geometry as both the triplet state and the singlet. Since the open-shell singlet in a single-determinant KS picture is an equal mixture of the triplet and the correct singlet state, the required singlet excitation energy can be extracted from these two calculations and used to shift the onset of the computed spectra.

As the Ru-CO distance is increased, we find the expected decrease in π* energy but overshooting the gas phase value and reaching a value lower than the gas phase reference. We ascribe this to charge-transfer from the metal to screen the charge on the molecule due to the removal of a half electron from the C 1*s*. Indeed, we find very similar total charge on CO for the ground state and the half core-hole (HCH) state up to ∼3 Å and a neutral ground state and ∼+0.1 charged HCH state beyond that. In the gas phase, the molecule in the TP-DFT approximation would have a +0.5 charge which means that even at 6 Å around 0.4 electronic charge has been transferred from the metal to screen the core-hole. This is supported by the low intensity of the π* peak at the longer distances since this state becomes partially occupied by the charge-transfer. We regard these effects as artifacts of the fixed half-core-hole in this approach when applied to cluster models and using Gaussian basis sets. These effects still exist for TP-DFT applied to periodic systems using plane wave (above) or numerical grid^7^ expansions of the wave functions.

### Ground-state response theory (TD-DFT)

**FIG. 7.**
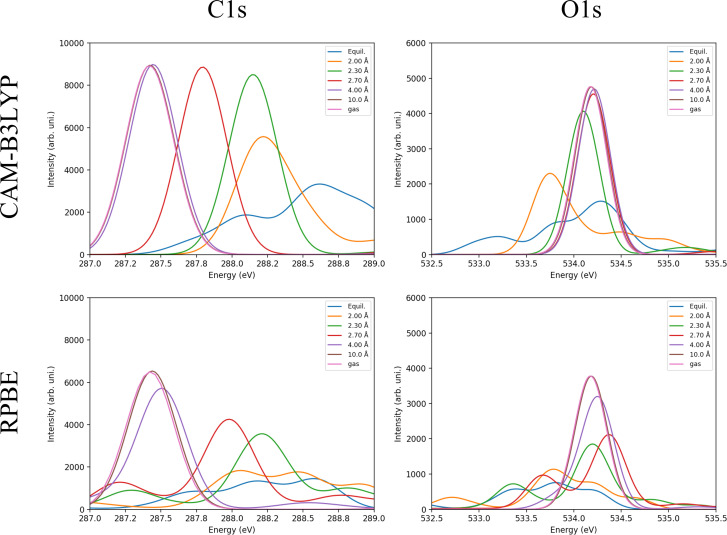
Carbon (C1s) and Oxygen (O1s) *K*-edge XAS calculated with linear-response TD-DFT for RPBE and CAM-B3LYP exchange-correlation functionals using the Ru_64_ cluster. OBS: The excitation energies in all calculated spectra were shifted to match experimental data.

Here, we summarize linear response TD-DFT XAS in Fig. 7 using the spectra obtained with RPBE and CAM-B3LYP functionals for the Ru_64_ cluster models. In the supplementary material, we include the spectra for all different combinations of exchange-correlation functionals and cluster sizes together with a more detailed discussion of the cluster size convergence and the choice of functional. However as a summary, we observe a convergence in the spectra for a cluster size of 31 Ru atoms when using a long-range-separated functional like CAM-B3LYP, while for the other functionals there is a noticeable difference between Ru_31_ and Ru_64_ XAS. However, Ru_31_ spectra for other functionals still show reasonable features compared to experiment and seem adequate for qualitative cases. Therefore, the evolution of the spectra seems more rapid and happens at shorter distances for RSF compared to global hybrid functionals, showing that while HF exchange at long-range beneficially leads to self-interaction cancelation for separated molecular states, the qualitatively good description of metallic screening at the GGA level is lost at long distances. Thus, the emergent metallic band structure formed with increasing cluster size is likely not as well described as with GGA approaches.

In general, comparing with the experimental spectra in Fig. 2, Fig. 7 shows that both RPBE and CAM-B3LYP for Ru_64_ correctly predict main trends for C and O K-edge XAS for the studied desorbing system. We observe the broader and more delocalized peak at chemisorbed states, with correct negative and positive shifts compared to the gas-phase for C1s and O1s, respectively. At 4.0 Å, the peak has already converged to the gas-phase limit for CAM-B3LYP, and it is almost converged for RPBE. Furthermore, C1s TD-DFT spectra give a similar quality peak evolution, but without suffering from artificial charge transfer or convergence to the wrong spin state. For O1s, the results of the cluster calculations seem qualitatively better than PBC-GGA that performed worse, even if we consider the singlet energy correction as discussed above.

The chemical shift is a key quantity in XAS, especially for comparison between calculated and experimental spectra, as DFT usually gives wrong absolute values of core excitations or ionizations, but, due to systematic error cancelations, can give quantitative chemical shifts. We summarize in Table I the calculated chemical shifts, i.e., the π* peak position relative to gas phase (positive for C and negative for O) for all functionals in PBC and cluster calculations. Since for some structures orbital interactions involving the π* and discrete states of the cluster make the peak position difficult to define, the position was defined as the average of all energies in the range (π* region) of the plotted spectra weighted by their respective intensities. The TD-DFT weighted average chemical shifts are stable across different cluster sizes (Table S2) and functionals, showing consistency even if the spectrum seems to have too many features. For both TP-DFT and TD-DFT the C K-edge shifts are overestimated while being underestimated for O, which in principle could be related to a high amount of stabilization of core-valence exchange interactions in the excited state. This would explain why TD-DFT/RPBE has better chemical shifts overall as the inclusion of exact exchange in the hybrid functionals can stabilize more the gas-phase excited state, where the π* orbitals are only located on CO, increasing the shift for C1s while decreasing it for O1s.

**TABLE I.** Chemisorbed-gas phase chemical shifts for carbon and oxygen (1s → π*) obtained from calculations and experiment related to the XA spectra of CO/Ru(0001). All values are in eV. The peaks for TD-DFT (Ru_64_) were calculated in both chemisorbed and gas phases as a weighted average from the energies in the respective spectrum range.

	**ΔC1s**	**ΔO1s**
Experiment	0.59^7^	−0.75^6^
RPBE/PBC	0.74	0.10
B3LYP	0.91	−0.16
CAM-B3LYP	1.12	−0.19
CAM-100%	1.10	−0.01
PBE0	1.01	−0.21
RPBE	0.71	−0.50

Furthermore, having in mind the good performance of linear-response TD-DFT and with the constant improvement in efficiency of excited-state wave function methods, one can even imagine that this approach could be extended to higher-level methods, like equation of motion coupled-cluster (EOM-CC)^83–85^ or the algebraic diagrammatic construction (ADC).^86^

## CONCLUSIONS

Calculations using TP-DFT with periodic boundary conditions and TD-DFT with cluster models were carried out and compared with x-ray absorption spectroscopy studies of CO desorption from a Ru(0001) surface. A set of five well-known exchange-correlation functionals were applied, and the cluster calculations were compared to standard periodic boundary condition calculations using the TP-DFT/RPBE approach. It was shown that clusters with sizes between 31 and 64 ruthenium atoms are feasible to treat and large enough to capture the most important effects around the chemical bond of interest when employing a combination of triple and double-zeta quality basis sets. While for C 1*s* XAS the PBC TP-DFT/GGA calculations yielded a description of the spectra evolution upon CO desorption, describing well the broadening and intensity ratios of the peaks for chemisorbed and non-chemisorbed structures and the chemical shift relative to the gas phase, the cluster models described better the evolution of O 1*s* spectra compared to experiment while still showing good results for C 1*s*. Although in most cases the C1s chemical shift is overestimated while the O1s is underestimated, all TD-DFT cluster calculations showed the correct trends when compared to experiment. Therefore, we conclude that finite-size cluster models may be used in conjunction with linear response methods to calculate core-excited transitions among transient species in an adsorbate–surface system. They can be a useful way to simulate spectra for adsorbates on systems where it is important to have a better description of the chemical bond, with emphasis on situations where the bond is not well defined (e.g., desorption processes), and we can make use of more sophisticated methods that are not available for PBC calculations. We also believe that a next and feasible step is to make use of higher-level wave function methods that could improve even more the calculated results.

We note, finally, that the here studied CO desorption presents particular challenges to the TP-DFT approach with a half core-hole. This is due to the low-lying π*, which can easily accept a screening charge leading to the observed loss of intensity and slight overshoot compared to the gas phase. In addition, the large exchange interaction between the π* and the unpaired spin in the 1*s* orbital results in a significant singlet-triplet split. This results in a too low π* excitation energy in a single-determinant formalism unless explicitly corrected for by either correcting the energy by calculating both the mixed single-determinant state and the correct triplet or by explicitly calculating the singlet as in the linear-response TDDFT calculations performed here. For systems without low-lying acceptor valence states, these effects can be expected to be much less significant and thus the TP-DFT also provide a reliable picture of the spectroscopic evolution as molecules desorb.

## SUPPLEMENTARY MATERIAL

The supplementary material contains potential energy surfaces (PES), example input file for ORCA cluster calculations, Ru custom basis set, natural transition orbitals (NTO) and XAS spectra for all clusters and functionals, PBC computational details, and XYZ coordinate files for all cluster calculations.

## Data Availability

The data that support the findings of this study are available within the article and its supplementary material.
